# Home-Made Cost Effective Preservation Buffer Is a Better Alternative to Commercial Preservation Methods for Microbiome Research

**DOI:** 10.3389/fmicb.2017.00102

**Published:** 2017-01-31

**Authors:** Sebastian Menke, Mark A. F. Gillingham, Kerstin Wilhelm, Simone Sommer

**Affiliations:** Institute of Evolutionary Ecology and Conservation Genomics, University of UlmUlm, Germany

**Keywords:** gut microbiome, fecal samples, sample preservation, swabs, buffer, freezing, sheep, next-generation sequencing

## Abstract

The investigation of wildlife gastrointestinal microbiomes by next-generation sequencing approaches is a growing field in microbial ecology and conservation. Such studies often face difficulties in sample preservation if neither freezing facilities nor liquid nitrogen (LQN) are readily available. Thus, in order to prevent microbial community changes because of bacterial growth after sampling, preservation buffers need to be applied to samples. However, the amount of microbial community variation attributable to the different preservation treatments and potentially affecting biological interpretation is hardly known. Here, we sampled feces of 11 sheep (*Ovis aries* sp.) by using swabs and analyzed the effect of air-drying, an inexpensive self-made nucleic acid preservation buffer (NAP), DNA/RNA Shield™, and RNA*later*®, each together with freezing (for 10 days) or storing at room temperature (for 10 days) prior to 16S rRNA gene high-throughput sequencing to determine bacterial communities. Results revealed that the proportions of operational taxonomic units (OTUs) belonging to a bacterial phylum were affected by the preservation treatments, and that alpha diversities [observed OTUs, Shannon index, and phylogenetic diversity (PD)] were lower in all preservation treatments than in samples taken by forensic swabs and immediately frozen which is considered as the favored preservation treatment in the absence of any logistic constraints. Overall, NAP had better preservation qualities than RNA*later*® and DNA/RNA Shield™ making this self-made buffer a valuable solution in wildlife microbiome studies.

## Introduction

Recent advances in high-throughput sequencing, computational techniques, and new bioinformatics tools have tremendously increased our knowledge about the beneficial role of symbiotic gut microbes in host nutrition, the immune system, and social interactions, and the negative implications of microbiome disorders promoting human diseases (Kau et al., 2011; Cho and Blaser, 2012; Althani et al., 2016; O'Doherty et al., 2016). Recently, this fast growing field of research has expanded into wildlife of which relatively little is known with regard to their gut microbiomes. Indeed, gut microbiome research into non-model species for which such information has previously been lacking is becoming increasingly popular and highlights important aspects with respect to animal health and conservation (Bahrndorff et al., 2016; Stumpf et al., 2016).

Typically, gut microbiomes are studied by sequencing phylogenetically important fragments of the 16S ribosomal RNA gene amplified from fecal DNA isolates by using a single primer combination. Since these primers amplify across bacterial taxa, this simple, and cost-effective approach provides ideal data sets for microbial community analyses (Soergel et al., 2012). After initial quality filtering, the obtained reads are clustered according to a similarity threshold, are compared with bacterial databases for assignment of taxonomy (DeSantis et al., 2006; Quast et al., 2013), and together with the information of the abundance per bacterial taxon, are written into a so-called table of operational taxonomic units (OTU). Subsequently, the effect of intrinsic or extrinsic factors on the microbial community and related diversity measures is investigated. Because many of these measures integrate information on bacterial diversity, phylogeny, and abundance per taxon, changes in bacterial communities attributable to inadequate storage of the initial fecal sample will bias results and subsequent biological conclusions.

An optimal starting point for such gut microbiome studies is the sufficient amount of fresh uncontaminated fecal sample from which DNA is immediately isolated. If this is not possible, then fresh samples should be immediately frozen until laboratory analyses can be carried out (Wu et al., 2010; Choo et al., 2015; Fouhy et al., 2015; Hale et al., 2015). By freezing samples, bacterial growth is inhibited, and the isolation of DNA and the amplification of target genes are not impeded by inhibitors, as is the case for some preservation media (Nechvatal et al., 2008). This is, however, often not feasible under field conditions, especially in research projects sampling wildlife in remote areas in which neither freezing facilities, nor liquid nitrogen (LQN) are readily available.

Several preservation media are currently available promising the conservation of sample characteristics from the time point of fecal collection until the isolation of DNA and the even more sensitive RNA. DNA/RNA Shield™ and RNA*later*® are frequently used as preservation media to preserve DNA and RNA in a variety of sample types. Because of their cost, cheaper alternatives are also frequently used such as the air-drying of specific swabs or the use of ethanol (Guo et al., 2016). The latter, however, has disadvantages because of its characterization as “dangerous goods” causing restrictions during international transport by plane. In other studies, research groups have devised their own formula for a preservation medium such as the homemade nucleic acid preservation (NAP) buffer (Camacho-Sanchez et al., 2013).

Here, we used sterile swabs to sample feces from 11 sheep and handled samples according to various preservation treatments for a time period of 10 days before DNA isolation: (1) forensic swabs (frozen/not frozen), (2) NAP (frozen/not frozen), (3) DNA/RNA Shield™ (frozen/not frozen), and (4) RNA*later*® (frozen/not frozen). Swabs are extremely practical tools for sampling bacteria because they are sterile, can be easily stored in 2 ml Eppendorf tubes, and most importantly, do not cause harm to the host species. Nevertheless, the investigation of gut microbiomes based on fecal swab samples, which are usually small in sample amount, might require good preservation treatments, because microbial communities might quickly diverge from their original state due to a strong effect of atmospheric oxygen on the bacterial community (Menke et al., 2015; Tedjo et al., 2015). We considered the “forensic swabs/frozen” treatment as the control treatment, since this would be the favored preservation treatment in the absence of constraints associated with field conditions and transport restrictions. We investigated whether preservation treatments affected characterization of sheep gut microbial communities and their alpha and beta diversities and further assessed the consistency of sequencing results based on replicate samples within preservation treatments. To the best of our knowledge, this is the only study that has investigated the effect of preservation media in combination with freezing/not freezing treatments on gut microbiome samples from both multiple individuals and replicates within an individual.

## Methods

### Treatments of fecal samples

We collected fecal samples from 11 sheep (*Ovis aries* sp.) kept on a private meadow in Baden-Wuerttemberg, Germany. Fecal samples were immediately thoroughly mixed in a plastic bag to obtain a homogeneous fecal sample. To evaluate the effects of various preservation treatments (forensic swabs, NAP buffer, DNA/RNA Shield™, and RNA*later*®) and storage conditions (room temperature, freezing), we took eight swabs from each fecal sample. Two forensic swabs (Sarstedt, Nuembrecht, Germany) were placed in 2 ml Eppendorf tubes without preservation medium. One was immediately frozen at −20°C, and the other stored at room temperature. Forensic swabs have a ventilation membrane that ensures an air-drying process while protecting against contamination. Furthermore, six fecal samples were taken with FLOQSwabs™ (Copan Flock Technologies, Brescia, Italy). Two of them were preserved in 2 ml Eppendorf tubes containing 600 μl homemade NAP medium (NAP; Camacho-Sanchez et al., 2013), two were preserved in 600 μl RNA*later*®, and two were stored in 600 μl DNA /RNA Shield™ (Zymo Research, Freiburg, Germany). FLOQSwabs are designed in a way that they release the entire sample into the preservation medium. From each preservation treatment, one swab-containing tube was frozen at −20°C after buffer incubation for 4 h, whereas the second swab was stored in the preservation buffer at room temperature. DNA was extracted from all samples after 10 days of storage under the respective storage condition. We considered the forensic swab/frozen treatment as the control treatment for all subsequent analyses.

To investigate the within-sample consistency and thus the effect of preservation buffer independently of variation between individuals, we took 41 swabs for one out of the 11 sheep (individual *R*). Out of these, five forensic swabs were air-dried (forensic swabs/frozen samples were not available for individual *R*), 12 swabs were stored in NAP (6 kept at room temperature, 6 kept frozen until extraction 10 days later), 12 swabs in DNA/RNA Shield™ (6 at room temperature, 6 frozen), and 12 swabs in RNA*later*® (6 at room temperature, 6 frozen). Finally, to control for cross-contamination, we sequenced eight blank samples [NAP (3), DNA/RNA Shield™ (3), or water (2)] which were randomly placed among real samples during DNA extraction and polymerase chain reaction (PCR) amplification.

### Microbiome DNA purification

The preservation media tested in this study required various treatments prior to DNA extraction. Because NAP and RNA*later*® have similar densities to those of the bacterial cells that were released from the swab into solution, it is difficult to re-pellet the cells via centrifugation. Furthermore, RNA*later*® can interfere with the DNA extraction process (Athanasio et al., 2016). To remove NAP and RNA*later*® from our samples, we diluted them by adding equal volumes of ice-cold phosphate-buffered saline (PBS) before centrifugation at 6000 g for 15 min as suggested in the manual (Life Technologies, 2011) and discarded the supernatant. DNA/RNA Shield™ has a more water-like density and can be used without reagent removal in most DNA purification kits; therefore, we did not pretreat these samples. The air-dried forensic swabs were soaked in 1 ml InhibitEx buffer from the QIAamp® Fast DNA Stool Mini Kit (Qiagen, Hilden, Germany). The pelleted material (NAP, RNA*later*®) and the dissolved material (DNA/RNA Shield™) were mixed with 1 ml InhibitEx buffer and homogenized with ceramic beads for 2 × 3 min on a SpeedMill (Analytik Jena, Germany). Thereafter, we followed the manufacturer's protocol for DNA extraction from stools for pathogen detection.

### PCR amplification, library preparation, and high-throughput sequencing

The extracted DNA was amplified with the universal bacterial primers 515F (5′-GTGCCAGCMGCCGCGGTAA-3′) and 806R (5′-GGACTACHVGGGTWTCTAAT-3′) to amplify a 291-bp fragment of the hypervariable V4 region of the 16S rRNA gene (Caporaso et al., 2010, 2011). Therefore, we followed the 4-primer amplicon tagging scheme of Fluidigm (Access Array™ System for Illumina Sequencing Systems, ©Fluidigm Corporation) in which tagged target specific primers (CS1-TS-515F and CS2-TS-806R) were combined with sample-specific primer pairs that contain a barcoding sequence and the adaptor sequences used by the Illumina sequencing systems. We added four random bases to our forward primers to avoid errors during cluster identification because of the high similarity of bases across all amplicons in the cluster identification cycles. We used the chemistry of Fluidigm with the initial amplicon PCR followed by a second barcoding PCR.

The initial 10 μl PCR volume contained 3–5 ng extracted DNA, 0.5 units FastStart Taq DNA Polymerase (Roche Applied Science, Mannheim, Germany), 1x PCR buffer, 4.5 mM MgCl_2_, 250 μM each dNTP, 0.5 μM primers, and 5% dimethylsulfoxide (DMSO). PCR was carried out with an activation step at 95°C for 4 min followed by 30 cycles at 95°C for 30 s, 60°C for 30 s, 72°C for 45 s, and a final elongation at 72°C for 10 min. The barcoding PCR volume (20 μl) contained 2 μl initial PCR product, 1 unit FastStart Taq DNA Polymerase, 1x PCR buffer, 4.5 mM MgCl_2_, 200 μM each dNTP, and 80 nM per barcode primer. PCR conditions were the same as before, but only 10 cycles were performed. Amplifications were quantified by UV/VIS spectroscopy on the Xpose (Trinean, Gentbrugge, Belgium) and samples were pooled to equimolar amounts of DNA. The library was prepared as recommended by Illumina (Miseq Reagent Kit v2—Reagent Preparation Guide) and was loaded at 7.5 p.m. on a MiSeq flowcell with 10% PhiX spiked in. Paired-end sequencing was performed over 2 × 251 cycles.

### Bioinformatics

To prepare reads for bacterial community analyses in QIIME [version 1.9.1; (Caporaso et al., 2010)] and phyloseq (McMurdie and Holmes, 2013), we carried out the following steps using default parameters unless stated otherwise. We (a) merged paired-end reads (*multiple_join_paired_ends.py* script in QIIME), (b) applied a quality threshold of *q* = 30 and a percentage of bases that must have this quality with *p* = 75 [*fastq_quality_filter.py* script in fastx-toolkit (FASTX-Toolkit)^1^], (c) converted fastq files into fasta files (*fastq_to_fasta.py* script in fastx-toolkit), and (d) cut primers by using cutadapt (Martin, 2011). The resulting reads were then used as a starting point for further analyses in QIIME, such as chimera checking, OTU-clustering, filtering of non-bacterial DNA, and calculation of alpha and beta diversities. Chimera checking was carried out by using USEARCH (Edgar et al., 2011) against the rep_set/97_otus.fasta (Greengenes version 13.5.) file (*identify_chimeric_seqs.py* script) of the Greengenes database (http://greengenes.lbl.gov,). We then applied an open reference OTU-clustering approach (*pick_open_reference_otus.py* script) with a similarity threshold of 0.97 also against the Greengenes database, again by using USEARCH (usearch61; Edgar, 2010), and taxonomy was assigned by using the RDP classifier (Wang et al., 2007). Subsequently, all non-bacterial DNA was removed from the dataset (*filter_taxa_from_otu_table.py* script) by using the corresponding identifiers (k_Eukaryota, c_Chloroplast, f_Mitochondria, k_Archaea).

### Statistical analysis

#### Effect of DNA yield on sequencing results

We tested the effect of preservation treatment on DNA yield using a generalized least-square (GLS) model. The response variable was log transformed DNA yield plus one and the explanatory variable was preservation treatment (“forensic swabs/frozen,” “forensic swabs/not frozen,” “NAP/frozen,” “NAP/not frozen,” “DNA/RNA Shield™/frozen,” “DNA/RNA Shield™/not frozen,” “RNA*later*®/frozen,” or “RNA*later*®/not frozen”). Heterogeneity between preservation treatments was controlled for (Zuur et al., 2009). We investigated whether sequencing depth differed according to DNA yield and preservation treatment using a linear mixed effects model (LMM) (Zuur et al., 2009; Pinheiro et al., 2016). The response variable was log transformed number of sequences and the explanatory variables were preservation treatment and DNA yield. Both the response variable and DNA yield were scaled. (i.e., each value was subtracted by the population mean and divided by the standard deviation of the population). The random factor was sheep identity. Model selection was achieved through information-theoretic (I–T) model selection (Burnham et al., 2002). All possible candidate models were constructed by using the predictor variables described above. Akaike's Information Criterion adjusted for small sample sizes (AICc) and AICc weights (ω) were used to assess the relative strength of support for models (Burnham et al., 2002; Johnson and Omland, 2004).

#### Effect of preservation treatments on alpha diversity measurements

To investigate the effects of preservation treatments on the proportion of OTUs belonging to the five most abundant phyla (*Firmicutes, Bacteroidetes, Verrucomicrobia, Proteobacteria, Spirochaetes*) and the proportion of OTUs belonging to the remaining phyla, we used a generalized linear mixed model (GLMM) with a binomial distribution using the lme4 package in R (Bates et al., 2015). We included the preservation treatment (“forensic swabs/frozen,” “forensic swabs/not frozen,” “NAP/frozen,” “NAP/not frozen,” “DNA/RNA Shield™/frozen,” “DNA/RNA Shield™/not frozen,” “RNA*later*®/frozen,” or “RNA*later*®/not frozen”) as an explanatory variable. The random factor was sheep identity. Again, model selection was achieved through information-theoretic (I–T) model selection (Burnham et al., 2002). Akaike's Information Criterion adjusted for small sample sizes (AICc) and AICc weights (ω) were used to assess the relative strength of support for models (Burnham et al., 2002; Johnson and Omland, 2004). To estimate the relative deviation in the proportion of OTUs belonging to a phylum between preservation treatments, we plotted the parameter estimates of GLMM models (with the control treatment “forensic swabs/frozen” as the intercept). Furthermore, we estimated the effect size odds ratio (OR; Nakagawa and Cuthill, 2007) to measure the deviation in the proportion of OTUs belonging to a phylum within a preservation treatment relative to the control treatment. The odds ratio measures the odds that an OTU occurs given that the sample is preserved in the investigated preservation treatment or in the control preservation treatment (an *OR* = 1 indicates no difference in the odds between the investigated preservation treatment or the control treatment; an *OR* >1 indicates an increase in the odds in the investigated preservation treatment; and an *OR* <1 indicates a decrease in the odds in the investigated preservation treatment).

To compare the effect of the various preservation treatments on bacterial diversities controlling for sequencing depth, we calculated three alpha diversity indices (“observed OTUs” (i.e., simply the abundance of OTUs), “Shannon diversity” Spellerberg and Fedor, 2003, and “phylogenetic diversity” (PD, Faith and Baker, 2007) for each gut microbiome sample. All measures of alpha diversity were scaled to facilitate the comparison of the effect of preservation treatment between alpha diversity measures. For statistical testing, we used general additive mixed models (GAMM) with a Gaussian distribution, an identity link function, and maximum-likelihood (ML) estimation by using the “mgcv” package in R (Wood, 2006). The preservation treatment (“forensic swabs/frozen,” “forensic swabs/not frozen,” “NAP/frozen,” “NAP/not frozen,” “DNA/RNA Shield™/frozen,” “DNA/RNA Shield™/not frozen,” “RNA*later*®/frozen,” or “RNA*later*®/not frozen”) and the number of sequences were entered as explanatory variables. The latter was included as a smoother term, and the maximum effective degrees of freedom (e.d.f), which determines the amount of smoothing, was limited to five. Sheep identity was used as a random factor, and we controlled for heterogeneity between preservation media and freezing treatments (Zuur et al., 2009). Model selection was achieved through information-theoretic (I–T) model selection as described above (Burnham et al., 2002). Also as above, we plotted the parameter estimates of GAMM models, and we calculated ORs within a preservation treatment relative to the control treatment (forensic swabs/frozen) as an effect size. Odds ratios can be calculated when an explanatory variable is continuous; however, in this case, ORs vary with the units of measurement (Nakagawa and Cuthill, 2007). Since the explanatory variables were scaled, the relative effect of preservation treatment on alpha diversity between different measures can be compared, but ORs are not an indication of the absolute effect of preservation treatments on individual alpha diversity measures.

#### Effect of preservation treatments on beta diversity

To compare the effect of the various preservation treatments on beta diversity, we calculated the weighted and unweighted UniFrac metric (Lozupone and Knight, 2005; Lozupone et al., 2011), which includes information on bacterial phylogeny, by using the R package “phyloseq.” We created a distance matrix including all individuals and a matrix with only the individual *R*. We tested the impact of individual sheep (including all individuals) and preservation treatment on weighted and unweighted UniFrac distance matrices by using a PERMANOVA approach [adonis function in R package “vegan,” (Oksanen et al., 2014)]. We used weighted as well as unweighted UniFrac metrics to test whether preservation methods had a stronger effect on relative abundances than on presence/absence of OTUs. Model selection was achieved through information-theoretic (I–T) model selection as described above (Burnham et al., 2002). We also report the amount of variation explained by each explanatory variable as *R*^2^ as defined in the adonis function in the R package “vegan.”

#### Ethics approval statement

Sheep from this study are privately held by Reinhold Wilhelm. Holding of these animals is approved by the ministry for nutrition, agriculture and forestry (AELF) in Wertingen (site master data number: 097731130105). Samples were collected after natural and voluntary defecation. Therefore, an ethics approval statement is not required for our study.

## Results

### Relationship between preservation treatment, DNA yield, and sequencing depth

**Figure 1:**
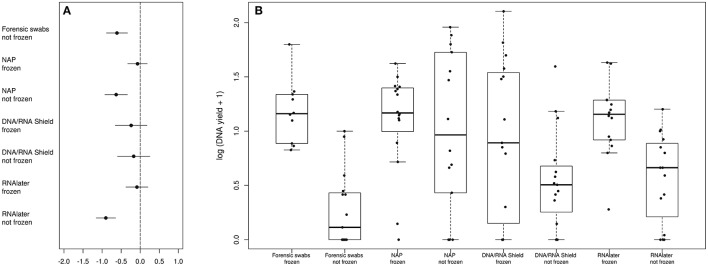
**Differences in DNA-yield between preservation treatments. (A)** Parameter estimates of the GLS models of DNA extraction yield according to preservation treatment. **(B)** Log(DNA-yield +1) for each sample of a respective preservation treatment.

DNA yield differed between preservation treatments (ΔAICc = 34.21, AICc ω = 1, Supplementary Table 1, Figure 1). Samples that had been frozen prior to DNA purification resulted in higher concentrations than non-frozen samples in all treatments except in the DNA/RNA Shield™ treatments (Figure 1). Model selection supported a negative relationship between sequencing depth and DNA yield (ΔAICc = 7.13, Supplementary Table 1, Supplementary Figure 1). Model selection also supported differences in sequencing depth between preservation treatments (ΔAICc = 8.44, Supplementary Figure 1). However, only “DNA/RNA Shield™/not frozen” had a significantly higher sequencing depth when compared to the control treatment (Supplementary Figure 1).

### Raw data of sequencing runs

We successfully sequenced the bacterial 16S rRNA gene in 114 out of 122 fecal samples of sheep (Supplementary Data 1, Supplementary Table 2) resulting in 3,681,449 reads after the merging of paired-end reads. After all bioinformatic steps had been carried out, the final OTU table was built upon 2,575,354 reads ranging from 7101 to 185,518 reads per individual (mean = 22,591, *sd* = 17,555). Noteworthy, “RNA*later*®/frozen” was the only preservation treatment for which no drop-out at sequencing occurred. Sequences obtained from blank samples contained only a few sequences (mean: 504.5, range: 114–1573), which were dominated by the bacterial phyla Proteobacteria and Actinobacteria, most likely due to bacterial contamination present in the extraction kits used (Salter et al., 2014). For an OTU found in the blanks (extraction and PCR blanks), the maximum proportion of reads in a sheep sample was 0.04% (and the median maximum for each OTU present in a blank sample found in a sheep sample was 0.00004%; Supplementary Data 2). Therefore, contamination from buffers as well as cross-contamination is very unlikely to have any discernible effect on the results of sheep samples.

### Relative abundance of bacterial phyla

**Figure 2:**
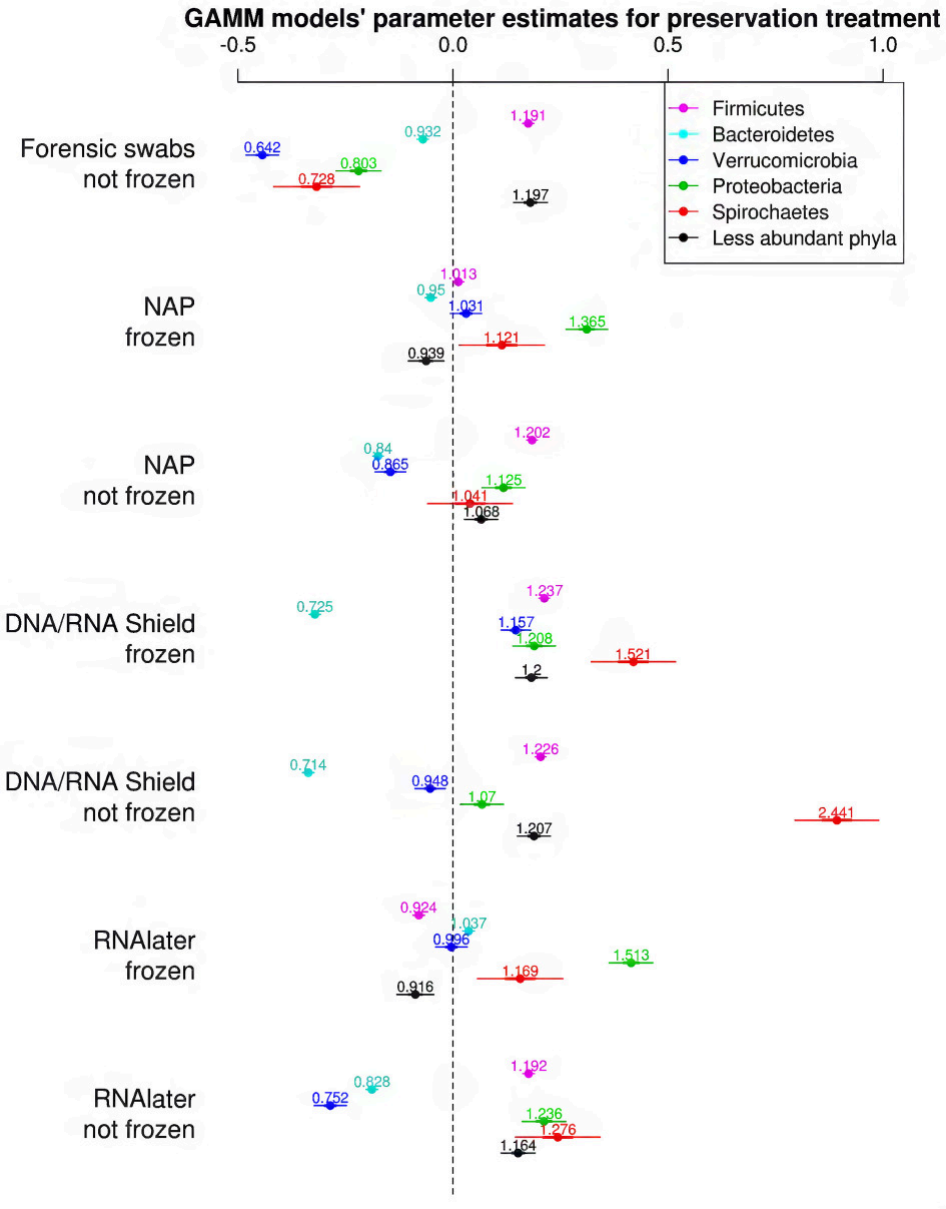
**Parameter estimates (points), 95% confidence intervals (thin lines), and standard deviation (thick lines) from GAMMs modeling the effect of preservation treatment on the proportion of OTUs belonging to the bacterial phyla Firmicutes, Bacteroidetes, Verrucomicrobia, Proteobacteria, Spirochaetes, and the remaining less abundant phyla**. The intercept was the control treatment (forensic swaps immediately frozen) of the samples. The dashed line represents the intercept, and a parameter estimate from a preservation treatment with 95% confidence intervals that do not overlap this dashed line represents a significant deviation from the control treatment. The response variable and the explanatory variable “number of sequences“ were scaled allowing a relative comparison of the effect of preservation buffers between measures of alpha diversity (but this means that absolute values of the intercept and number of sequences are not represented here). Numbers above parameter estimates are odds ratios that represent the deviation in the proportion of OTUs belonging to a phylum within a preservation treatment relative to the control treatment (Forensic swabs/frozen).

The bacterial phyla with the highest proportion of OTUs across all preservation treatments were Firmicutes [mean 54.60, 95% CI (51.34, 57.12)], Bacteroidetes [mean 33.45, 95% CI (31.34, 35.91)], Verrucomicrobia [mean 3.28, 95% CI (2.79, 3.74)], Proteobacteria [mean 2.49, 95% CI (1.96, 3.22)], and Spirochaetes [mean 2.04, 95% CI (1.12, 3.10)] (Table 1; see Supplementary Data 3 for the highest possible taxonomic resolution). Model selection revealed strong support for an effect of preservation treatments on the proportion of OTUs belonging to Firmicutes, Bacteroidetes, Verrucomicrobia, Proteobacteria, Spirochaetes, and other less abundant phyla (Figure 2, Table 2). For the three most common bacterial phyla that dominated the bacterial community (Firmicutes, Bacteroidetes, and Verrucomicrobia), “NAP/frozen” was the preservation treatment that deviated the least from the control treatment (Figure 2). Indeed, for Firmicutes and Verrucomicrobia, 95% confidence intervals of the parameter estimates overlapped zero, and ORs were close to 1 for all three phyla (Figure 2). The preservation treatment “NAP/frozen” led to a higher proportion of OTUs belonging to the phyla Proteobacteria and Spirochaetes and to a lower proportion belonging to the less abundant phyla (Figure 2). However, overall, the deviation in the proportion of OTUs was less strong for samples preserved in “NAP/frozen” relative to the other treatments investigated in this study. The only other preservation treatment that was comparable with “NAP/frozen” was the preservation treatment “RNA*later*®/frozen.” The proportion of OTUs from the dominant phylum Firmicutes, however, deviated considerably more than in the “NAP/frozen” treatment relative to the control (*OR* = 0.924 relative to *OR* = 1.013 for NAP/frozen; Figure 2), and the deviation relative to the control for Proteobacteria, Spirochaetes, and other less abundant phyla was much stronger (Figure 2). Regarding treatments in which freezing was not applied, the preservation for which the deviation in the proportion of OTUs belonging to the phylum was the least strong relative to the control was “NAP/not frozen,” with OR values ranging between 0.84 and 1.20 (a much narrower range than all other non-frozen treatments, Figure 2).

**Table 1:** **Ten most abundant bacterial phyla of the sheep gut microbiome**.

	**Forensic swabs**		**NAP**		**DNA/RNA Shield**™		**RNA*later*^®^**	
	**Frozen**	**Not frozen**	**Frozen**	**Not frozen**	**Frozen**	**Not frozen**	**Frozen**	**Not frozen**
Firmicutes	60.03 ± 0.03	54.93 ± 0.02	50.08 ± 0.03	54.43 ± 0.03	56.92 ± 0.02	54.06 ± 0.02	52.20 ± 0.03	54.15 ± 0.03
Bacteroidetes	31.90 ± 0.04	35.77 ± 0.04	35.93 ± 0.05	33.74 ± 0.04	29.99 ± 0.04	30.59 ± 0.04	36.34 ± 0.05	33.35 ± 0.04
Verrucomicrobia	3.16 ± 0.03	2.22 ± 0.02	3.97 ± 0.04	3.25 ± 0.03	3.45 ± 0.03	3.64 ± 0.04	3.79 ± 0.03	2.72 ± 0.02
Proteobacteria	1.23 ± 0.01	1.72 ± 0.02	3.36 ± 0.04	2.58 ± 0.03	2.80 ± 0.03	2.69 ± 0.03	2.56 ± 0.03	2.96 ± 0.03
Spirochaetes	0.32 ± 0.01	1.22 ± 0.02	2.13 ± 0.04	1.88 ± 0.03	2.71 ± 0.05	4.40 ± 0.09	1.30 ± 0.02	2.34 ± 0.04
Planctomycetes	0.68 ± 0.04	0.23 ± 0.01	0.71 ± 0.05	0.59 ± 0.04	0.71 ± 0.05	0.78 ± 0.05	0.71 ± 0.05	0.54 ± 0.04
Cyanobacteria	0.52 ± 0.01	0.77 ± 0.01	0.42 ± 0.01	0.50 ± 0.01	0.53 ± 0.01	0.52 ± 0.01	0.37 ± 0.00	0.72 ± 0.01
Tenericutes	0.31 ± 0.00	0.60 ± 0.01	0.31 ± 0.00	0.37 ± 0.00	0.40 ± 0.00	0.52 ± 0.00	0.29 ± 0.00	0.47 ± 0.00
Lentisphaerae	0.51 ± 0.01	0.61 ± 0.01	0.20 ± 0.00	0.24 ± 0.00	0.19 ± 0.00	0.25 ± 0.01	0.25 ± 0.01	0.43 ± 0.01
Fibrobacteres	0.29 ± 0.02	0.13 ± 0.01	0.20 ± 0.01	0.29 ± 0.02	0.18 ± 0.01	0.17 ± 0.01	0.15 ± 0.01	0.25 ± 0.02

*Numbers represent the mean ± standard deviation of the respective phylum within a preservation treatment (here we did not control for sheep individual and sequencing depth)*.

**Table 2:** **Model selection of GLMMs of the proportion of OTUs from the phyla Firmicutes (A), Bacteroidetes (B), Verrucomicrobia (C), Proteobacteria (D), Spirochaetes (E), and other less abundant phyla (F) according to preservation treatment; showing number of parameters (K), log-likelihood (logLik), AICc of the models, change in AICc compared with the best-ranked model (ΔAICc), and Akaike model weights (ω)**.

**Model Rank**	**Preservation treatment**	**K**	**logLik**	**AICc**	**ΔAICc**	**ω**
**(A) FIRMICUTES**						
1	+	9	−7969.479	15958.69	0	1
2		2	−10838.249	21680.61	5721.92	<0.001
**(B) BACTEROIDETES**						
1	+	9	−6952.420	13922.84	0	1
2		2	−11369.165	22742.33	8819.49	<0.001
**(C) VERRUCOMICROBIA**						
1	+	9	−3482.662	6985.054	0	1
2		2	−4702.280	9408.669	2423.61	<0.001
**(D) PROTEOBACTERIA**						
1	+	9	−2667.006	5353.742	0	1
2		2	−3707.030	7418.168	2064.43	<0.001
**(E) SPIROCHAETES**						
1	+	9	−2170.888	4361.507	0	1
2		2	−6081.280	12166.67	7805.16	<0.001
**(F) OTHER LESS ABUNDANT PHYLA**						
1	+	9	−1494.506	3007.011	0	1
2		2	−1783.630	3571.261	564.249	<0.001

*Sheep identity was entered as a random factor. Directions of the effects of preservation treatments are presented in Figure 2*.

### Alpha diversity

When controlling for sequencing depth, model selection revealed strong support for an effect of preservation treatment on all three alpha diversity measures (Table 3: a. Observed OTUs: ΔAICc = 8.13, AICc ω = 0.983, b. Shannon diversity: ΔAICc = 17.05, AICc ω = 0.999, c. PD: ΔAICc = 7.47, AICc ω = 0.977).

**Table 3:** **Model selection of GAMMs of (A) observed OTUs, (B) Shannon diversity, and (C) phylogenetic diversity (PD) according to sequencing depth (smoother term) and preservation treatment; showing number of parameters (k), log-likelihood (logLik), AICc of the models, change in AICc compared to the best-ranked model (ΔAICc), and Akaike model weights (ω)**.

**Model Rank**	**Sequences**	**Preservation treatment**	**K**	**logLik**	**AICc**	**ΔAICc**	**ω**
**(A) OBSERVED OTUs**							
1	+	+	19	−13.783	73.651	0	0.983
2	+		12	−27.344	81.777	8.13	0.017
3			10	−133.280	288.697	215.05	<0.001
4		+	17	−125.742	291.858	218.21	<0.001
**(B) SHANNON DIVERSITY**							
1	+	+	19	−102.407	250.900	0	0.999
2		+	17	−113.052	266.478	15.58	<0.001
3	+		12	−120.429	267.948	17.05	<0.001
4			10	−130.845	283.826	32.93	<0.001
**(C) PHYLOGENETIC DIVERSITY**							
1	+	+	19	−6.327	58.739	0	0.977
2	+		12	−19.558	66.206	7.47	0.023
3			10	−120.148	262.432	203.69	<0.001
4		+	17	−112.520	265.414	206.68	<0.001

**Figure 3:**
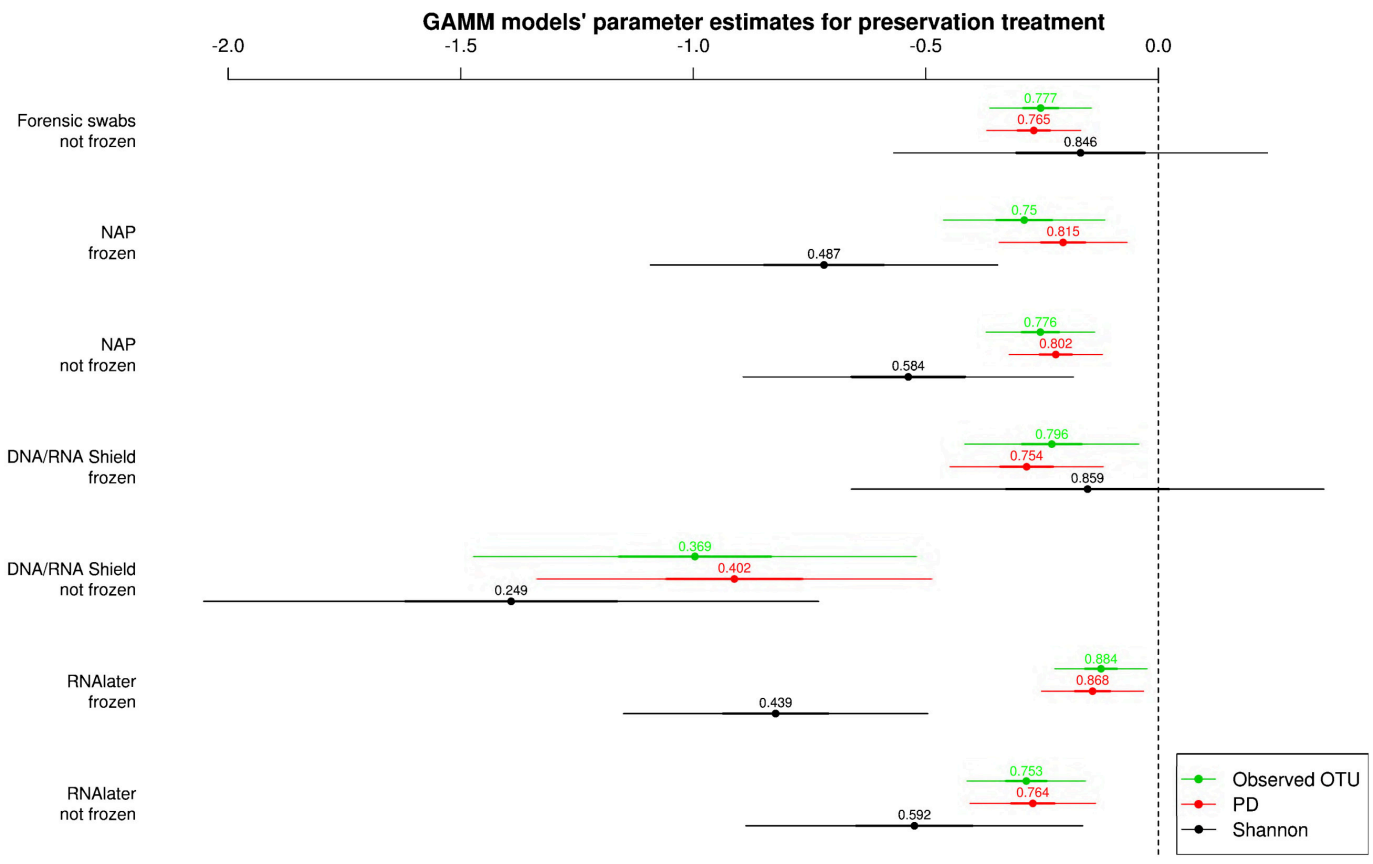
**Parameter estimates (points), 95% confidence intervals (thin lines), and standard deviation (thick lines) from GAMMs modeling the effect of preservation treatment on Observed OTUs, PD, and Shannon diversity controlling for sequencing depth**. The intercept was the control treatment (Forensic swabs/frozen).of the samples. The dashed line represents the intercept, and a parameter estimate from a preservation treatment with 95% confidence intervals that do not overlap this dashed line represents a significant deviation from the control treatment. The response variable and the explanatory variable “number of sequences“ were scaled allowing a relative comparison of the effect of preservation buffers between measures of alpha diversity (but this means that absolute values of the intercept and number of sequences are not represented here). Numbers above parameter estimates are odds ratios that represent the deviation in the alpha diversity within a preservation treatment relative to the control treatment (forensic swabs/frozen).

*Sheep identity was entered as a random factor. Directions of the effects of preservation treatments are presented in Figure 3*.

All alpha diversity measures for each preservation treatment (Figure 3) were lower than the alpha diversities of the control treatment (intercept in Figure 3). Only the Shannon diversity of “forensic swabs/not frozen” and “DNA/RNA Shield™/frozen” had 95% CI of the estimate overlapping zero (Figure 3). The negative effect of preservation treatments on alpha diversity relative to “forensic swabs/frozen” was stronger for “DNA/RNA Shield™/not frozen” than for all the other preservation treatments (Figure 3). The latter suggests that bacteria in “DNA/RNA Shield™/not frozen” were poorly preserved relative to all other preservation treatments. However, for all of the remaining preservation treatments, alpha diversity was very similar (Figure 3).

### Microbiome variation within and between individuals

**Figure 4:**
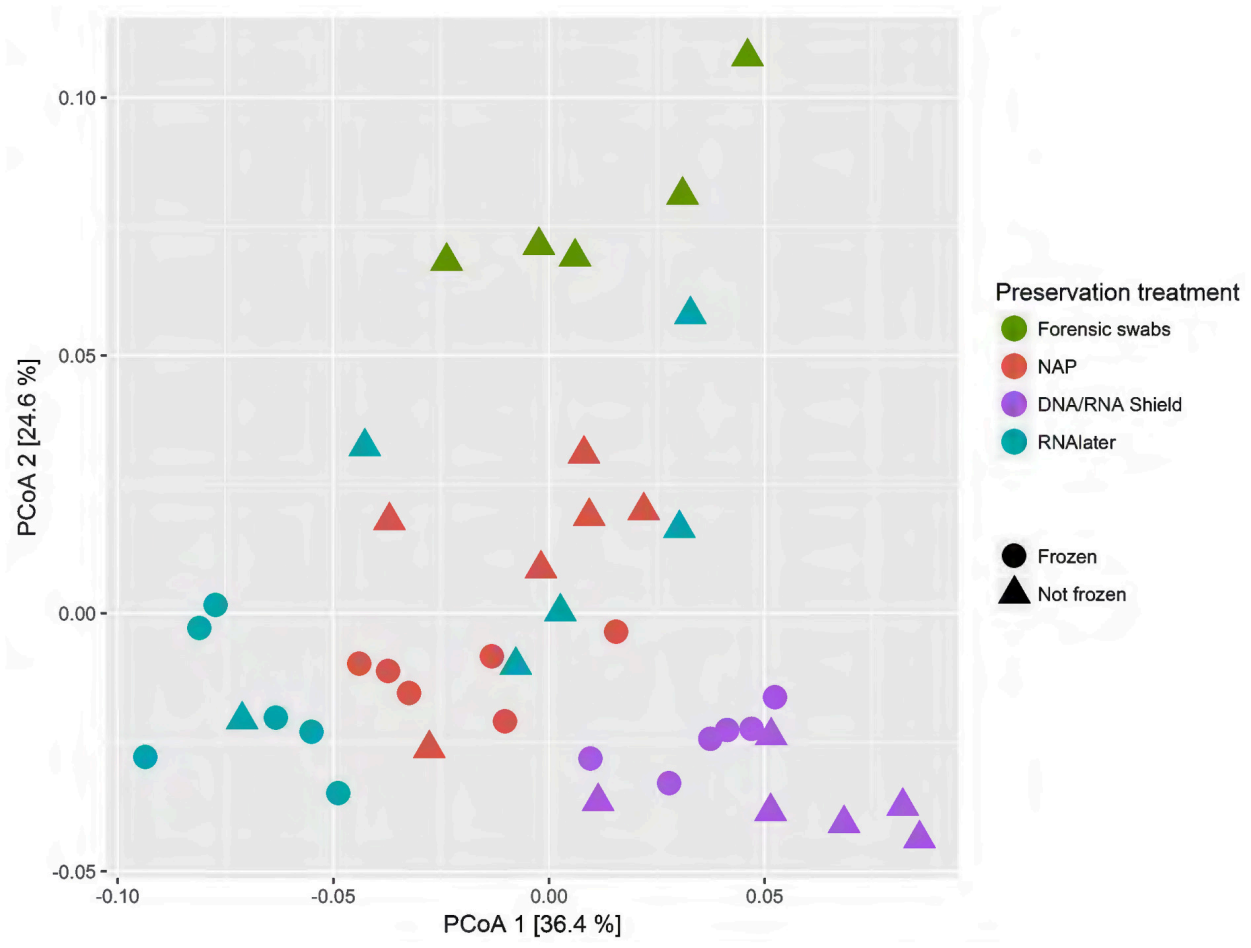
**Principal coordinate analysis (PCoA) of samples of sheep individual R according to preservation treatment based on the weighted-UniFrac metric**. The x-axis and y-axis together explain 58.1% of the total variation. Most of the variation was explained by preservation treatment (ΔAICc = 22.06, AICc ω = 1).

**Figure 5:**
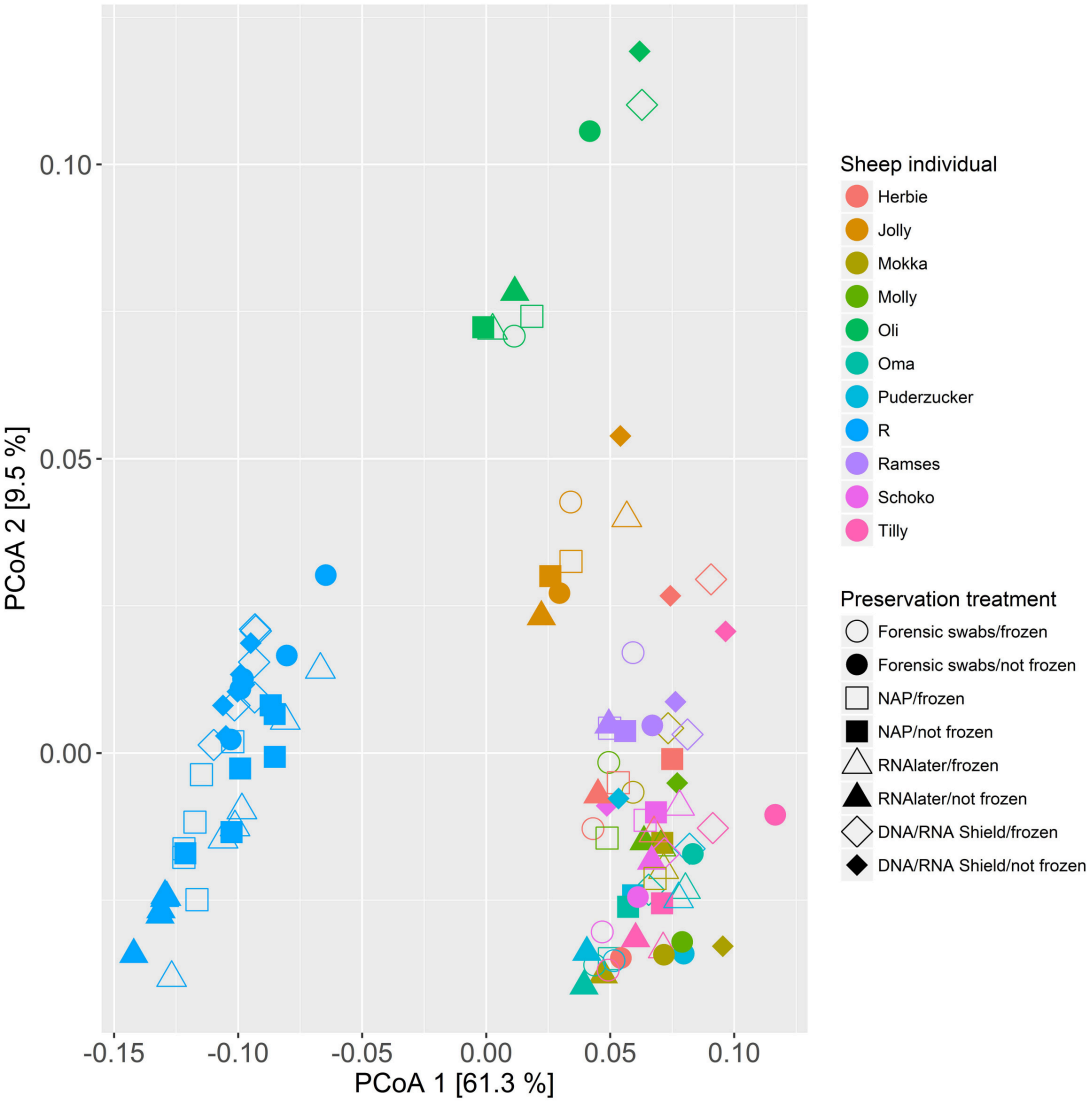
**Principal coordinate analysis (PCoA) of samples of all sheep individuals according to preservation treatment based on the weighted-UniFrac metric**. The x-axis and y-axis together explain 70.8% of the total variation. Most of the variation was explained by sheep individual (ΔAICc = 189.10; *R*^2^ = 0.77), followed by preservation treatment (ΔAICc = 43.89; *R*^2^ = 0.10).

When we tested for within-individual consistency between preservation treatments based on replicates of individual *R* (Figure 4), the results revealed that samples clustered according to preservation treatment (Table 4A: ΔAICc = 22.06, AICc ω = 1). However, when investigating the microbiome variation based on the weighted UniFrac metric including all sheep individuals, model selection revealed stronger support for an effect of sheep identity than for preservation treatment (Figure 5, Table 4B). Indeed, most of the variation was explained by sheep identity (Table 4B: ΔAICc = 189.10; *R*^2^ = 0.77), followed by preservation treatment (Table 4B: ΔAICc = 43.89; *R*^2^ = 0.10). The same analysis based on the unweighted UniFrac metric revealed that sheep individuals still explained most of the variation (ΔAICc = 21.59; *R*^2^ = 0.32; Supplementary Table 3). Interestingly, there was little support for an effect of preservation treatment (Supplementary Table 3; ΔAICc = −9.03; *R*^2^ = 0.06), suggesting a weak effect of preservation treatment on the absence/presence of an OTU.

**Table 4:** **Model selection of PERMANOVA models based on the weighted UniFrac metric according to sheep identity and preservation treatment; showing number of parameters (k), log-likelihood (logLik), AICc of the models, change in AICc compared to the best-ranked model (ΔAICc), and Akaike model weights (ω)**.

**Model Rank**	**Sheep identity**	**Preservation treatment**	**K**	**logLik**	**AICc**	**ΔAICc**	**ω**
**(A) DATASET WITH INDIVIDUAL** ***R*** **ONLY**							
1		+	8	81.882	−143.263	0	1
2			2	62.762	−121.208	22.06	<0.001
**(B) DATASET WITH ALL INDIVIDUALS**							
1	+	+	19	191.610	−337.135	0	1
2	+		12	160.166	−293.244	43.89	<0.001
3			2	76.07298	−148.038	189.10	<0.001
4		+	9	82.955	−146.180	190.96	<0.001

## Discussion

In this study, we assessed the effect of different preservation treatments on the composition of the sheep gut bacterial community, alpha and beta diversities, and within-treatment consistency. Swabs were used for fecal sampling and were air-dried (in case of the forensic swabs), or stored in self-made NAP-buffer, DNA/RNA Shield™, or RNA*later*®, and either immediately frozen or kept at room temperature for 10 days after sampling until DNA extraction. The aim was to infer the suitability of these preservation treatments for field studies in which freezing or immediate transportation to a laboratory for high-throughput sequencing is not possible. We found that, when freezing facilities are available, forensic swabs remained the best preservation method. However, when the latter is not possible, preserving samples in the homemade NAP buffer gives the best results overall.

Preservation treatments affected DNA yield and sequencing depth, but the later did not affect the results of our statistical tests because we accounted for sequencing depth in our models. Samples with a lower DNA yield resulted in a higher sequencing depth, which is most certainly due to the fact that the accuracy of DNA measurement is lower when DNA yield is low, leading to an underestimation of the true yield when normalizing samples for sequencing. Our data does not enable us to distinguish whether differences in DNA yield are due to the extraction methods associated with the buffer or the buffer in itself. However, centrifugation during extraction for RNA*later*® and NAP buffer are unavoidable since cells have the same density for these reagents. Anybody using these reagents will therefore be obliged to follow these steps, so we strongly feel that whether differences are due to different extraction methods or buffer is marginal in this case. Extraction yield was lower for all treatments that were not frozen except of “DNA/RNA Shield™/frozen” suggesting that the latter buffer preserved DNA integrity best when samples were kept at room temperatures.

The alpha diversity measurements obtained from “forensic swabs/not frozen” were very similar to samples stored in “DNA/RNA Shield™/frozen,” making both the least different relative to the control treatment. However, the proportions of OTUs belonging to a bacterial phylum strongly deviated from the control treatment revealing the importance of freezing during the sample preservation of forensic swab samples. Our results based on the unweighted UniFrac matrix suggests that air-drying of forensic swab samples for short periods (10 days in this study) prior to freezing or to the isolation of genetic material is permissible for studies which only focus on the presence/absence of bacterial taxa but not their relative abundances. However, longer drying periods should be avoided since differences in sample characteristics and climatic conditions at the sampling sites could lead to more dramatic bacterial community changes (Menke et al., 2015).

We confirmed that whether samples were frozen or not frozen affected microbial communities, e.g., the proportion of OTUs belonging to a bacterial phylum, which has been shown also in other studies (Bahl et al., 2012; Flores et al., 2015). This is in contrast to studies that found no differences according to freezing treatment which could be due to the fact that our non-frozen samples were kept at room temperature for a longer time period (10 days) than samples of those studies (24 h–3 days; Dominianni et al., 2014; Tedjo et al., 2015). Therefore, freezing regardless of the preservation treatment, whenever possible, should be applied. Furthermore, all preservation treatments had lower alpha diversities relative to frozen forensic swabs. Thus, forensic swabs are recommended for field work when freezing after a short drying period is possible at the field station.

All preservation treatments involving a commercial buffer (DNA/RNA Shield™ and RNA*later*®) had a strong effect on the proportion of OTUs belonging to a phylum. DNA/RNA Shield™ performed worse than RNA*later*®, both when samples were frozen and not frozen, and alpha diversities for “DNA/RNA Shield™/not frozen” were much lower compared to all other preservation treatments. This might be due to the absence of very low abundant bacterial OTUs in the “DNA/RNA Shield™/not frozen” samples compared to the control because an overgrowth of specific bacterial taxa could not be observed (Supplementary Data 3). However, surprisingly, NAP performed better than all other treatments, particularly when freezing was applied, since the three most abundant phyla (Firmicutes, Bacteroidetes, and Verrucomicrobia) had almost equal proportions of OTUs relative to the control treatment. Furthermore, the alpha diversities of samples preserved in NAP buffer were comparable with other treatments, regardless of freezing. These results are promising as they confirm that the self-made NAP buffer (Camacho-Sanchez et al., 2013) is a cheap alternative to the expensive RNA*later*® and DNA/RNA Shield™, particularly when freezing facilities are not readily available. Although the NAP buffer recipe is straight-forward, the preservation quality depends on the accuracy of the person preparing it in the laboratory. This could lead to a potential bias when samples within a project were stored in homemade buffers prepared by different people. Nevertheless, this type of error can easily be eliminated when researchers are aware of this issue.

Overall, a stronger effect of preservation treatment was seen on Shannon diversity than on observed OTUs and PD, and also on the weighted than on to the unweighted UniFrac metric. This was due to fact that preservation treatments had a stronger effect on the relative abundances rather than on the absence/presence of taxa (as demonstrated by the effect of preservation treatment on the proportion of OTUs belonging to a phylum, and on the weighted and unweighted UniFrac metric). Thus, care should be taken when comparing values from different preservation treatments (Tedjo et al., 2015) and especially when those values are based on metrics that incorporate relative abundances of taxa such as the Shannon index. On the positive side, despite the effect of preservation treatment, most variation in sheep microbiomes was explained by the individual, as was shown in other studies (Dominianni et al., 2014; Hale et al., 2015; Song et al., 2016) suggesting strong consistency in sampling within the method applied. In addition, when a study is only interested in presence/absence data, the effect of preservation treatment was even lower. Thus, useful comparisons can be made, even when freezing facilities are not readily available, as long as the same preservation method is applied to all samples within a study.

## Conclusion

In the absence of any logistical constraints the immediate freezing of forensic swab without buffer is the favored preservation treatment. For situations in which a preservation buffer is required, our results confirm the preservation abilities of commercial buffers for gut microbiome studies, but also reveal that the self-made NAP buffer gives superior results. NAP buffer efficiency in stabilizing bacterial communities in swab-collected fecal matter worked better than commercially available preservation media, even when freezing is not available, making this low-price self-made preservation medium a good alternative for low-budget wildlife microbiome studies in remote areas. Furthermore, the fact that NAP is not considered as “dangerous goods” makes this preservation medium valuable in situations in which samples have to be globally shipped.

## Data accessibility

Data available from the Dryad Digital Repository: http://dx.doi.org/10.5061/dryad.5rk06.

## Author contributions

Conceived and designed the project: MG, SS, and KW. Performed the experiments: MG, KW. Bioinformatics and statistics: MG, SM. Writing of manuscript: MG, SM, SS, KW.

## Funding

Financial support was provided by DFG grants to SM (DFG SO 428/10-1) as well as to MG (DFG Gi 1065/2-1).

### Conflict of interest statement

The authors declare that the research was conducted in the absence of any commercial or financial relationships that could be construed as a potential conflict of interest.

## Footnotes

^1^FASTX-Toolkit http://hannonlab.cshl.edu/fastx_toolkit/index.html by Hannon Lab.
