# Supplementary material for: Home-Made Cost Effective Preservation Buffer Is a Better Alternative to Commercial Preservation Methods for Microbiome Research
Source: Front Microbiol. 2017 Jan 31;8:102. doi: 10.3389/fmicb.2017.00102 (PMC5281576; doi:10.3389/fmicb.2017.00102)
Supplement: Supplementary file 1 [file Table1.DOCX]

**Supplementary Table 1:** Model selection of a) GLS models of DNA extraction yield according to preservation treatment and b) LMM models of sequencing depth according to preservation treatment and DNA extraction yield; showing number of parameters (k), log-likelihood (logLik), AICc of the models, change in AICc compared to the best-ranked model (ΔAICc), and Akaike model weights (ω). For GLS models heterogeneity between preservation treatments was controlled for. For LMM models sheep identity was entered as a random factor. Directions of the effects of explanatory variables are presented in Figure 1 and Supplementary Figure 1.

| **Model Rank** | **DNA extraction yield** | **Preservation treatment** | **K** | **logLik** | **AICc** | **ΔAICc** | **ω** |
| --- | --- | --- | --- | --- | --- | --- | --- |
| **a. DNA extraction yield** | | | | | | | |
| 1 |  | + | 16 | -68.432 | 174.471 | 0 | 1.000 |
| 2 |  |  | 9 | -94.473 | 208.676 | 34.21 | <0.001 |
| **b. Sequencing depth** | | | | | | | |
| 1 | + | + | 18 | -124.224 | 291.648 | 0 | 0.956 |
| 2 |  | + | 17 | -129.203 | 298.781 | 7.13 | 0.027 |
| 3 | + |  | 11 | -137.747 | 300.083 | 8.44 | 0.014 |
| 4 |  |  | 10 | -140.733 | 303.601 | 11.95 | 0.002 |
