# Supplementary material for: Home-Made Cost Effective Preservation Buffer Is a Better Alternative to Commercial Preservation Methods for Microbiome Research
Source: Front Microbiol. 2017 Jan 31;8:102. doi: 10.3389/fmicb.2017.00102 (PMC5281576; doi:10.3389/fmicb.2017.00102)
Supplement: Supplementary file 2 [file Table2.DOCX]

**Supplementary Table 2: Sheep gut microbiome experimental design.** Samples which failed at sequencing are represented with a zero.

|  | **Forensic swabs** | | **NAP** | | **DNA/RNA Shield** | | **RNA*later*®** | |
| --- | --- | --- | --- | --- | --- | --- | --- | --- |
|  | **Frozen** | **Not-frozen** | **Frozen** | **Not-frozen** | **Frozen** | **Not-frozen** | **Frozen** | **Not-frozen** |
| **Herbie** | 1 | 1 | 1 | 1 | 1 | 1 | 1 | 1 |
| **Jolly** | 1 | 1 | 1 | 1 | 0 | 1 | 1 | 1 |
| **Mokka** | 1 | 1 | 1 | 1 | 1 | 1 | 1 | 1 |
| **Molly** | 1 | 1 | 1 | 0 | 1 | 0 | 1 | 1 |
| **Oli** | 1 | 1 | 1 | 1 | 1 | 1 | 1 | 1 |
| **Oma** | 1 | 1 | 1 | 1 | 0 | 1 | 1 | 1 |
| **Puderzucker** | 1 | 1 | 0 | 1 | 1 | 1 | 1 | 1 |
| **Ramses** | 1 | 1 | 1 | 1 | 1 | 1 | 1 | 0 |
| **Schoko** | 1 | 1 | 1 | 1 | 1 | 1 | 1 | 1 |
| **Tilly** | 1 | 1 | 0 | 1 | 1 | 1 | 1 | 1 |
| ***R*** | NA | 5 | 6 | 6 | 6 | 6 | 6 | 6 |
