# Supplementary material for: Home-Made Cost Effective Preservation Buffer Is a Better Alternative to Commercial Preservation Methods for Microbiome Research
Source: Front Microbiol. 2017 Jan 31;8:102. doi: 10.3389/fmicb.2017.00102 (PMC5281576; doi:10.3389/fmicb.2017.00102)
Supplement: Supplementary file 3 [file Table3.DOCX]

**Supplementary Table 3:** Model selection of PERMANOVA models based on the unweighted UniFrac metric according to sheep identity and preservation treatment; showing number of parameters (k), log-likelihood (logLik), AICc of the models, change in AICc compared to the best-ranked model (ΔAICc), and Akaike model weights (ω).

| **Models** | **Sheep identity** | **Preservation treatment** | **K** | **logLik** | **AICc** | **ΔAICc** | **ω** |
| --- | --- | --- | --- | --- | --- | --- | --- |
| **a. Dataset with individual *R* only** | | | | | | | |
| **Mnull** |  |  | **2** | **-23.1513** | **50.6184** | **0** | **0.984** |
| **M1** |  | **+** | **8** | **-19.1983** | **58.8966** | **58.8966** | **0.016** |
| **b. Dataset with all individuals** | | | | | | | |
| **M2** | **+** |  | **12** | **-58.4066** | **143.902** | **0** | **0.989** |
| **M1** | **+** | **+** | **19** | **-53.4249** | **152.935** | **9.0327** | **0.011** |
| **Null** |  |  | **2** | **-80.6908** | **165.49** | **21.5876** | **2E-05** |
| **M3** |  | **+** | **9** | **-76.8826** | **173.496** | **29.5937** | **3.7E-07** |
