# Supplementary material for: Home-Made Cost Effective Preservation Buffer Is a Better Alternative to Commercial Preservation Methods for Microbiome Research
Source: Front Microbiol. 2017 Jan 31;8:102. doi: 10.3389/fmicb.2017.00102 (PMC5281576; doi:10.3389/fmicb.2017.00102)
Supplement: Supplementary file 7 [file Image1.PDF]

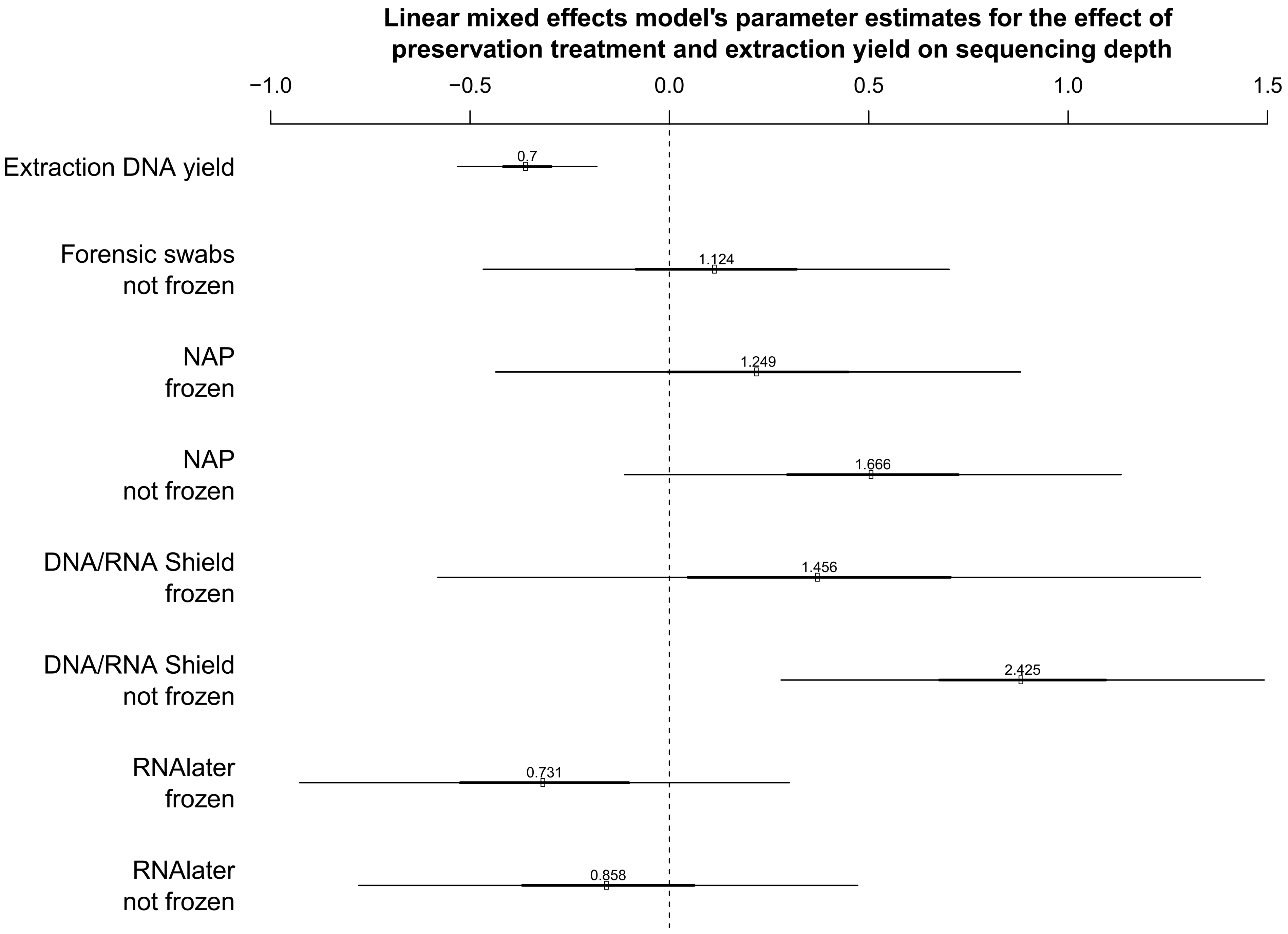

Figure S1: Parameter estimates (points), 95% confidence intervals (thin lines), and standard deviation (thick lines) from LMMs modeling the effect of preservation treatment and extraction yield on sequencing depth. The intercept was the control treatment (forensic swaps immediately frozen). The dashed line represents the intercept, and a parameter estimate with 95% confidence intervals that do not overlap this dashed line represents a significant deviation from the control treatment. The response variable and the explanatory variable "Extraction DNA yield" were scaled allowing a relative comparison of the effect of preservation buffers on sequencing depth (but this means that absolute values of the intercept and number of sequences are not represented here). Numbers above parameter estimates are odds ratios that represent the deviation in the sequencing depth within a preservation treatment relative to the control treatment (Forensic swabs/frozen).
